# I like the way you move: how animate motion affects visual attention in early human infancy

**DOI:** 10.3389/fnins.2024.1459550

**Published:** 2024-08-13

**Authors:** Marco Lunghi, Elisa Di Giorgio

**Affiliations:** ^1^Department of Developmental Psychology and Socialization, University of Padova, Padova, Italy; ^2^Department of Women’s and Children’s Health (SDB), University of Padova, Padova, Italy

**Keywords:** visual attention, animacy, biological motion, infants, social brain

## Abstract

The ability to detect animates (as compared with inanimates) rapidly is advantageous for human survival. Due to its relevance, not only the adult human brain has evolved specific neural mechanisms to discriminate animates, but it has been proposed that selection finely tuned the human visual attention system to prioritize visual cues that signal the presence of living things. Among them, animate motion—i.e., the motion of animate entities -, is one of the most powerful cues that triggers humans’ attention. From a developmental point of view, whether such specialization is inborn or acquired through experience is a fascinating research topic. This mini-review aims to summarize and discuss recent behavioral and electrophysiological research that suggests that animate motion has an attentional advantage in the first year of life starting from birth. Specifically, the rationale underlying this paper concerns how attention deployment is affected by animate motion conveyed both by the movement of a single dot and, also, when the single dot is embedded in a complex array, named biological motion. Overall, it will highlight the importance of both inborn predispositions to pay attention preferentially to animate motion, mainly supported by subcortical structures, and the exposure to certain experiences, shortly after birth, to drive the cortical attentional visual system to become the way it is in adults.

## 1 Introduction

To identify conspecifics and to differentiate them from other kinds of entities is of paramount adaptive value, because they feed us, interact (or at least they should) successfully with us, love us, and help us to hand down our genetic endowment (Bonatti et al., 2002). From an evolutionary point of view, it is plausible to hypothesize that we have inherited ancestrally-derived and highly conserved mechanisms, shared with other species (i.e., chicks, non-human primates), deputed to detect biologically relevant entities, such as prey, predators, and social companions. Indeed, it has been suggested that the human visual system is evolved to pay attention and to prioritize animates on the basis of some pictorial cues, such as the presence of a face, eye-gaze, and body structure. The *way they move* is another cue that attracts humans and non-humans’ visual attention toward animate entities.

Motion of living beings, here referred to *“life motion*” as conceptualized by Troje (2013), which includes any kind of visual stimulus that elicits the percept of something being alive, has specific features that are not shared with motion of inanimate objects, such as the capability to start from rest or to change speed and trajectory without external forces (i.e., self-propulsion). In Troje’s taxonomy, life motion is further differentiated between *extrinsic* and *intrinsic* motion. Extrinsic motion refers to *animate motion perception* elicited by motion of single dot or geometrical shapes (Heider and Simmel, 1944; Carey, 2009; Rutherford and Kuhlmeier, 2013; Scholl and Gao, 2013), whereas intrinsic motion refers to *biological motion* (BM) *perception* (Johansson, 1973).

Neuroscientific evidence and neuropsychological lesion studies (Battelli et al., 2003; Gilaie-Dotan et al., 2011) suggested that in the adult brain life motion detection is subserved by relatively specific set of both subcortical and cortical areas within the so called *Social Neural Network* (SNN) (Caramazza and Shelton, 1998; Blakemore et al., 2003; Schultz et al., 2005; Adolphs, 2009; Frith and Frith, 2010; Santos et al., 2010; Stosic et al., 2014). Not only, but it has been proposed that selection finely tuned the human visual attention system to prioritize visual cues that signal the presence of living things (New et al., 2007). Further, SNN atypicalities lead to impairments in animate (Rutherford et al., 2006) and BM motion perception (Federici et al., 2020) observed in Autism Spectrum Disorders (ASD, Sato and Uono, 2019).

Ontogenetically, whether such a level of neural and functional specialization for life motion detection found in adults is present from birth or is the result of developmental processes, is still an open question. Several behavioral studies demonstrated in human newborns the presence of inborn attentional predispositions toward visual cues of motion that trigger both animate and BM perception in adults (Simion et al., 2008; Bardi et al., 2011, 2015; Bidet-Ildei et al., 2014; Di Giorgio et al., 2017, 2021a). However, the missing piece of the puzzle concerns the experience-dependent or experience-independent nature of the mechanisms underlying such attentional biases, as well as their neural bases.

Starting from the evidence of the presence of specialized brain and attention systems in human adults, here we sought to overview and discuss the current state of the art on (i) the role of attentional predispositions in triggering visual attention toward both extrinsic (animate) and intrinsic (biological) motion at birth and in the first months of life, and (ii) the experience-independent or experience-dependent nature of the underpinning mechanisms thought to be involved in such attentional advantage.

## 2 Life motion in the adult brain: neural and functional specialization

Adults perceive animacy based on some basic visual cues of motion, such as self-propulsion, movement speed, contingency (Tremoulet and Feldman, 2000; Santos et al., 2008). Neuroimaging studies have revealed that the adult brain is equipped with a highly specialized neural network, the SNN, devoted to process such animate motion (Santos et al., 2010), that includes both subcortical—i.e., insula and amygdala—and cortical structures—i.e., the ventromedial prefrontal cortex (vmPFC), the superior temporal gyrus (STG) and sulcus (STS), the fusiform gyrus (FG) (Blakemore et al., 2003; Van Overwalle, 2009; Frith and Frith, 2010; Grill-Spector and Weiner, 2014). Similarly to what we known about non-human primates (Oram and Perrett, 1994; Tomonaga, 2001; Jellema et al., 2004; Jastorff et al., 2012), converging brain structural and connectivity evidence suggests that STS plays a key role in extrinsic and intrinsic motion processing (Schultz et al., 2005; Pavlova et al., 2006; Wheatley et al., 2007; Gobbini et al., 2011; Herrington et al., 2011; Pavlova, 2012; Sokolov et al., 2012; Yokoyama et al., 2021).

This brain specialization is coupled, at the behavioral and functional level, with an *attentional advantage* for stimuli that are characterized by animate motion and BM. According to the *animate-monitoring hypothesis* (New et al., 2007), humans’ visual attentional system has evolved to prioritize and monitor visual cues central to our survival. Despite some studies that have questioned this hypothesis (Hagen and Laeng, 2016; Cox et al., 2022), it has been shown that animates were detected more quickly and accurately, rather than inanimates, in different attentional tasks, such as attentional blink (Calvillo and Hawkins, 2016; Guerrero and Calvillo, 2016), inattentional blindness (Calvillo and Jackson, 2014), change blindness (New et al., 2007; Altman et al., 2016), and visual search (Abrams and Christ, 2003; Pratt et al., 2010).

Likewise, some evidence suggested that a similar attentional advantage is present also for BM (Chandler-Mather et al., 2020). BM elicits faster saccadic reaction (Bardi et al., 2015) and more accurate responses (Shi et al., 2010; Hirai et al., 2011) in a cueing paradigm when compared with other motion types, and it can be processed incidentally in a flanker task (Thornton and Vuong, 2004). Based on this and other results, some authors postulated the presence of an inborn mechanism sensitive to BM, namely *perceptual life detector* (Johnson, 2006; Troje and Westhoff, 2006).

From a developmental perspective, it is plausible to hypothesize that animate and BM perception has an early ontogenetic origin because in adults it is based on fast and automatic visual processing, which is mainly constrained by a collection of low-level visual motion cues. Whether similar attentional advantages found in adults for animate and BM are present at birth, as well as the experience-dependent or experience-independent nature of perceptual mechanisms postulated at the basis of it, are relevant and intriguing open questions.

## 3 How animate motion affects visual attention in newborns and infants

Controversy still exists about the origin of animate motion perception (Biro and Leslie, 2007; Rakison et al., 2008). Some authors have hypothesized that infants come into the world equipped with inborn domain-specific mechanisms (Leslie, 1995), otherwise referred as core-knowledge (Spelke and Kinzler, 2007), that allow them to be sensitive to visual cues of motion that characterize animates. Others suggested that domain-general inborn predispositions to such visual cues of motion might be a sort of building blocks to the development of animacy perception through learning mechanisms (cue-based bootstrapping model, Biro and Leslie, 2007).

To disentangle the issue, developmental studies have mainly focused on few-month-old infants’ ability to attribute goals, intentions and emotions based on the objects’ motion (Poulin-Dubois et al., 1996; Luo and Baillargeon, 2005; Biro et al., 2007; Luo et al., 2009; Mahajan and Woodward, 2009), named *intentional agency* (Carey, 2009). However, on which visual cues infants used to infer such an abstract concept from early on, that is animate motion perception, is much a relatively unexplored topic of research.

At the behavioral level, some studies suggested that animate motion captures infants’ visual attention. For instance, Träuble et al. (2014) showed that speed and direction changes by self-propulsion induce animacy perception in 7-month-old infants. Infants’ visual attention was not only attracted by single object events, but also by objects that move contingently as in the case of chasing events. It has been demonstrated that 5-and 12-month-old infants orient their attention to and attend preferentially toward the agent that initiates an interaction within a chasing event (Galazka and Nyström, 2016). Frankenhuis et al. (2013) demonstrated an attentional bias for chasing events based on visual cues of motion (acceleration, high turning rates, and attraction) and their combination (chasing) in 4-and 10-month-old infants. Further, using a change-detection paradigm, it has been shown that 11-month-old infants are able to detect changes faster to animate compared to inanimate stimuli (Hofrichter et al., 2021).

This behavioral evidence is further corroborated by the results obtained in an ERP study with infants of 9 months. By this age infants allocate more attentional resources to an object moving inanimately than an animate object as evidenced by the increased negativity in the fronto-central Nc component, a component that indexes general attentional arousal, demonstrating differential sensitivity to animate and inanimate motion (Kaduk et al., 2013).

All these studies concern infants of a few months with a certain degree of visual and social experience, leaving the question about the origin of such perception open. To the best of our knowledge, only two recent studies demonstrated newborns sensitive toward some low-level visual cues of motion that trigger animacy perception in adults, which are self-propulsion and speed changes without external forces. In a series of visual preference experiments, 2-day-old newborns orient their attention preferentially toward an object that started from rest (Di Giorgio et al., 2017), and changed its speed without external forces (Di Giorgio et al., 2021a), compared to an object that did not start to move or change speed by its own. Authors interpreted the presence of such inborn attentional biases as a sort of bootstrapping point to the development of animacy and agency perception found later during development, corroborating the cue-based bootstrapping model (Biro and Leslie, 2007), which emphasizes the interplay between such inborn predispositions and the role of experience and learning mechanisms during development.

As for other visual cues that indexed the presence of animate beings (i.e., faces and BM), these attentional biases are thought to be mainly guided by subcortical neural structures (the superior colliculi) which appear to contribute to the specialization of the brain cortical circuits that, during development, carry out more sophisticated social information processing. Broadly speaking, such inborn experience-independent biases not only cause newborns to orient reflexively their attention toward animate beings, but they may serve to bias voluntary cortical-mediated visual attention toward relevant stimuli over the first weeks and months of life to promote the typical progressive specialization of the SNN (Johnson, 2005; Johnson et al., 2015). It has been suggested that such a shift from reflexive-to-cortical-mediated visual mechanisms could be altered in ASD (Di Giorgio et al., 2021b), leading to a cascading effects on animate and BM perception atypicalities (Rutherford et al., 2006).

While recent findings on the currently elective animal model (domestic chicks) is very promising in shedding new light on neural and physiological mechanisms underlying inborn attentional biases (Lorenzi et al., 2017; Mayer et al., 2019; Rosa-Salva et al., 2021), in human newborns the neural signature of such biases remains almost speculative (see Buiatti et al., 2019).

## 4 How biological motion affects visual attention in newborns and infants

As shown so far, infants’ and newborns’ attention are triggered by animate motion conveyed by the movement of a single dot. Life motion detection may also be driven by more specific types of motions cues, such as when the relative motion of many dots in a complex point light display (PLD) are organized and perceived as a particular form of vertebrates’ motion such as a human walker (Johansson, 1973; Scholl and Gao, 2013). In a complex array of dots, each single dot has self-propelled motion and follows the acceleration and deceleration motion pattern. When these cues of motion are applied to dots that composed complex arrays as in the case of BM, adults are able to extract, simply analyzing motion, a lot of social information.

Historically, the first study that investigates sensitivity toward BM during development, through use of PLD, was done by Fox and McDaniel (1982) testing 4-and 6-month-old infants. Starting from this first evidence, a growing number of studies tried to understand the origin of the sensitivity to BM.

Following the evidence coming from newly hatched chicks that demonstrates visual preference for BM rather than other motion types in this species (Vallortigara et al., 2005), Simion et al. (2008) investigates whether also in human newborn this attentional bias was present. In a series of experiments, it has been shown that that even 3-day-old human newborns have a predisposition to selectively orient their visual attention toward BM when compared with other motion types (random or rigid motion, Simion et al., 2008; Bardi et al., 2011, 2015; Bidet-Ildei et al., 2014). These results seem to support the hypothesis of the presence of a perceptual life detector which allows newborns of different species to orient preferentially their visual attention toward the local information (i.e., the motion of the limbs of an animal in locomotion) vehiculated by BM (Johnson, 2006; Troje and Westhoff, 2006). As for the inborn biases for animate motion, it has been hypothesized that attentional biases for BM at birth should be considered as a predisposed and experience-independent perceptual mechanism supported by subcortical structures (Simion et al., 2008; Chang et al., 2018).

Based on this assumption, several authors tried to better define the developmental trajectory of the sensitivity toward BM. In a series of studies, Bertenthal et al. (1984, 1987), investigated infants’ ability to organize complex arrays of dots as a coherent figure. Five-month-old infants are able to discriminate PLD walker from the same stimulus with scramble spatial relationship or with perturbed local rigidity between joints, suggesting the emergence of sensitivity toward global information level of BM. This suggests that the visual system at birth can use visual information to prefer and discriminate between coherent displays but not to integrate the individual motion information to form the global percept of a PLD. The ability to process configural relations in BM must involve, therefore, a more specialized, late-developing, higher-level processing; hence requiring more visual experience and cortical maturation (Hirai and Senju, 2020).

This evidence, coupled with the ones by Fox and McDaniel (1982), supports the hypothesis that the second mechanism that would be responsible in recognizing and identifying agents based on configural information would not be inborn, but rather an experience-dependent mechanism that becomes functioning during development (Troje and Westhoff, 2006). The extraction of relevant social information would be supported by this second mechanism (Chang and Troje, 2009), that might be used to pay voluntary attention and to interpret other intentions.

From a neural point of view, fNIRS studies showed that the selective activation of rSTS for BM processing emerges around 7–8 months (Lisboa et al., 2020a,b). The same pattern of results was found also in a neurophysiological study with 8-month-infants, where a modulation of ERP component peaking around 200–300 ms post-stimulus onset have been observed only in the right hemisphere (Reid et al., 2006). More recently, Hirai and Hakuno (2022) demonstrated that even at 6 months of age infants are able to extract a coherent human figure from PLDs, showing a larger ERP component for BM rather than scrambled motion, peaking around 500–600 ms post-stimulus onset.

Starting from this state of the art, research has attempted to investigate the functional meaning of BM walking direction processing, contemporarily probing the hypothesis according to which the walking direction of legged vertebrates may produce an attentional advantage in processing relevant information (Bardi et al., 2015; Lunghi et al., 2019, 2020). In these studies, authors tested 3-and 6-months-old infants using a cueing paradigm (Posner, 1980) with BM as central cue. Behaviorally, shorter saccadic reaction time for the target that appears in congruent position to the walking direction of BM was found. At the neural level, 3-and 6-month-old infants showed a modulation of ERPs sensory component (P1) for congruent targets even before the oculomotor response, suggesting that already at 3 months BM elicits a covert orienting of attention toward relevant information (Lunghi et al., 2019).

Overall, these findings suggest that the inborn sensitivity to BM, supported by an experience-independent mechanism, should be considered as a bootstrapping point to the development of the ability of infants and adults to extract social information from motion. Behavioral results showed atypical visual orienting toward BM stimuli in newborns and 4-month-old infants at high-risk for ASD (Di Giorgio et al., 2016, 2021b), corroborating the idea of the alteration to such mechanisms early in life.

## 5 Conclusion and future directions

The studies reviewed here seem to corroborate the hypothesis that life motion that characterizes living beings modulates visual attention starting from birth. Humans and non-human newborns (Mascalzoni et al., 2010; Rosa-Salva et al., 2016; Lemaire et al., 2022; Rosa-Salva et al., 2023) orient their visual attention reflexively toward visual cues of motion that characterized animate motion and BM since birth.

As proposed by Frankenhuis and Barrett (2013), it is plausible that starting from birth, infants use a “*coarse attentional filter that navigates their attention toward social interactions.”* Such a filter is shared with other vertebrates, therefore phylogenetically old, and it is sensitive to general properties of both animate motion and BM regardless of their content. Only later during development, infants are able to infer the social meaning conveyed by such visual cues of motion, thanks to more sophisticated cognitive (and cortical-guided) mechanisms dedicated to comprehending goals and intentions.

Recent groundbreaking studies are telling us that for understanding how cognition originates and develops, and which is the role of experience, some answers can be found before birth, in fetuses (Reid, 2024). Fetuses show impressive perceptual capabilities in visual (Reid et al., 2017; Donovan et al., 2020), auditory (Vogelsang et al., 2023), and motor domains (Zoia et al., 2007), making them a promising model for future studies on the origin of knowledge.

If the presence of such bootstrapping inborn attentional biases is not questioned, the neural signatures underlying such biases remain unexplored. For instance, albeit based on indirect evidence in human newborns, such inborn attentional biases are thought to be mainly guided by a rapid and reflexive subcortical orienting mechanisms, and that, around 2–3 months of life, are being replaced by voluntary cortical-mediated visual attention mechanisms (Johnson, 2005). In line with the recent findings about the neural substrates underlying face detection in human newborns (Buiatti et al., 2019), future studies, taking advantage from non-invasive methods such as EEG or fNIRS, should investigate which are the neural pathways involved in animate and BM perception, mainly unexplored at birth as well as in the first years of life.

Finally, the study of the neural bases underlying animate and BM perception is important in typical-developing populations. But it is even more important in ASD high-risk populations, where the need for noninvasive and sensitive biomarkers reflecting altered key functional circuits is urgent. This would corroborate studies, currently controversial (Dawson et al., 2023), that suggest altered inborn attentional predispositions as potential predictive risk factors for ASD.
